# A self‐reinforced activatable photosensitizer prodrug enabling synergistic photodynamic and chemotherapy

**DOI:** 10.1002/smo.20240005

**Published:** 2024-06-14

**Authors:** Daipeng Huang, Jikai Yin, Yang Zou, Haiqiao Huang, Saran Long, Wen Sun, Jianjun Du, Jiangli Fan, Xiaojun Peng

**Affiliations:** ^1^ State Key Laboratory of Fine Chemicals Frontiers Science Center for Smart Materials Oriented Chemical Engineering Dalian University of Technology Dalian China; ^2^ College of Life Science Key Laboratory of Biotechnology and Bioresources Utilization of Ministry of Education Dalian Minzu University Dalian China; ^3^ College of Materials Science and Engineering State Key Laboratory of Fine Chemicals Shenzhen University Shenzhen China

**Keywords:** cell apoptosis, chemotherapy, glutathione, photodynamic therapy, prodrug

## Abstract

As a novel drug development paradigm, selective activation of prodrugs provides the potential for precise tumor chemotherapy, thereby presenting an opportunity for advancing cancer treatment. The combination of photodynamic therapy (PDT) and prodrug can enhance the therapeutic efficacy while simultaneously enabling real‐time monitoring of drug distribution and release. However, the tumor hypoxia microenvironment and the frequent high‐dose administration of prodrugs significantly impede therapeutic efficacy and escalate treatment‐related risks. Herein, a tumor microenvironment‐specific release prodrug is constructed, termed NBS‐2S‐5FU. Under the influence of glutathione (GSH), NBS‐2S‐5FU undergoes activation, leading to the release of photosensitizer NBS and chemotherapeutic agent 5‐FU derivatives. Under irradiation, NBS produces sufficient superoxide radical ($O_{2}^{-•}$) while 5‐FU derivatives inhibit DNA biosynthesis, thereby effectively suppressing tumor growth at low doses. Subsequent in vivo studies utilizing NBS‐2S‐5FU liposomes exhibit outstanding anti‐cancer effectiveness. This study highlights a promising direction for advancing combined prodrugs that integrate PDT and chemotherapy.

## Graphical abstract

An intelligent self‐reinforced photosensitizer prodrug (NBS‐2S‐5FU) based on GSH activation has been constructed, leading to a synergistic enhancement in photodynamic therapy and chemotherapy. This prodrug not only effectively inhibits DNA biosynthesis but also amplifies the photocytotoxicity of PDT, culminating in the induction of cell apoptosis. This pathway presents an attractive prospect for advancing combined prodrugs that integrate PDT and chemotherapy.

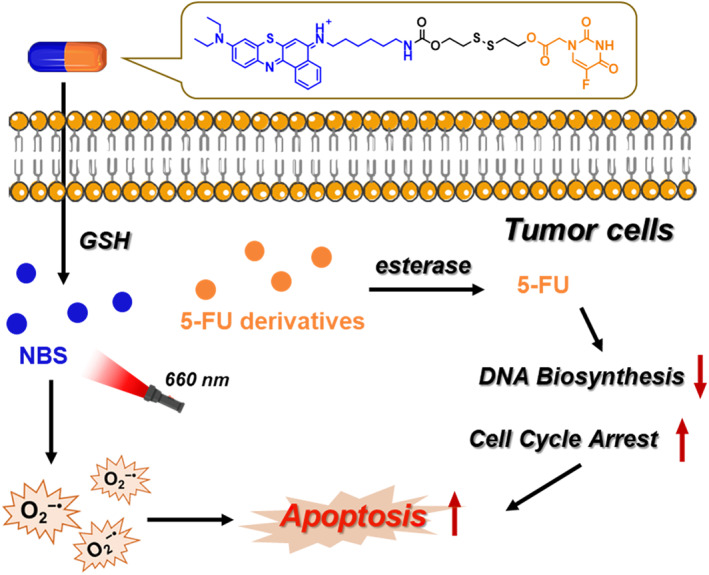

## 1 INTRODUCTION

Chemotherapy is widely regarded as one of the foremost methods in the clinical management of cancer, but the low tumor accumulation rate and frequent high‐dose use of conventional chemotherapy drugs severely limit their therapeutic effect, which may also have adverse effects on normal tissues.^[1]^ In recent years, selective activation prodrugs and multifunctional drug carrier materials have been designed and developed to achieve precise chemotherapy of tumor.^[2]^ Among them, prodrugs have gained attention because they are usually low‐toxic to normal cells and high‐toxic to tumor cells after specific activation.^[3]^ The excellent selectivity of prodrugs not only reduces cytotoxicity at off‐target sites but also minimizes the potential for high‐dose induced drug resistance.^[4]^

Nowadays, the chemotherapeutic agent 5‐fluorouracil (5‐FU) continues to serve as the cornerstone of chemotherapy drugs widely utilized in contemporary clinical practice.^[5]^ 5‐FU is a cell cycle‐specific drug that induces cell apoptosis by interfering with DNA biosynthesis. However, due to high side effects and short half‐life, fewer than half of patients benefit from 5‐FU‐based chemotherapy.^[6]^ The anti‐tumor efficiency of 5‐FU can be improved using it along with other drugs, or by developing 5‐FU derivatives to reduce side effects and drug metabolism rate.^[7]^ At present, most of the therapeutic prodrugs for 5‐FU are based on drug carrier materials, which have drawbacks such as complex synthesis processes and unknown biological safety.^[8]^ Small molecule prodrugs have inherent advantages such as easy production and industrialization. Therefore, the development of small molecule prodrugs based on 5‐FU is of great significance.

**FIGURE 1:**
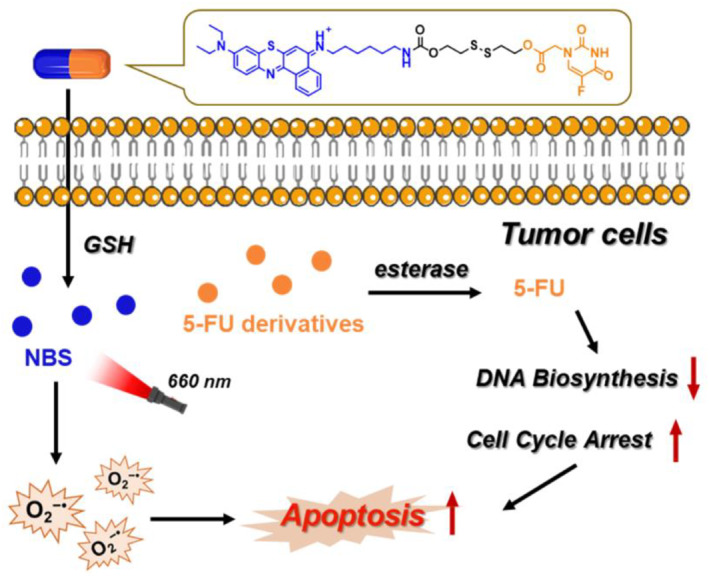
Schematic representation of NBS‐2S‐5FU utilizing GSH activation for synergistic enhancement of photodynamic therapy and chemotherapy.

Currently, photodynamic therapy (PDT) combined prodrugs have become very attractive in monitoring drug delivery and improving treatment efficacy,^[9]^ as controllable treatment sites and times can be achieved by controlling light irradiation.^[10]^ It has been reported that 1‐carboxylmethyl‐5‐fluorouracil (5‐FU acid) is beneficial for the photoactivity of photosensitizers in the PDT process.^[11]^ Meanwhile, considering the relatively higher concentration of glutathione (GSH) in tumor cells (approximately 10‐100‐fold) compared to normal cells and tissues,^[12]^ we have constructed a smart self‐reinforced activatable prodrug (abbreviated as NBS‐2S‐5FU) (Figure 1). In this prodrug, the type I PDT reagent sulfur‐substituted Nile Blue (NBS) is covalently linked to 5‐FU acid through disulfide bonds. After the disulfide bond is cleaved by GSH, the prodrug is converted into NBS through intramolecular cyclization, and 5‐FU derivatives are released (Figure S1). Under irradiation, NBS produces sufficient superoxide radical ($O_{2}^{-•}$) to effectively inhibit tumor growth, while 5‐FU derivatives can inhibit DNA biosynthesis, jointly enhancing the photocytotoxicity of PDT. The subsequent in vivo studies utilizing NBS‐2S‐5FU liposomes demonstrate outstanding efficacy in combating cancer. In short, NBS‐2S‐5FU demonstrates that the combination of chemotherapy and PDT with activatable prodrugs has the potential for tumor treatment, and low doses can efficiently kill tumors, which is of great significance for reducing drug resistance and evaluating the prognosis of cancer treatment.

## 2 RESULTS AND DISCUSSION

### 2.1 Synthesis and photophysical properties of NBS‐2S‐5FU

The multi‐step synthesis pathway of NBS‐2S‐5FU was depicted in Scheme S1 and the characterization data of NBS‐2S‐5FU were shown in supporting information (Figure S13‐S15).

**FIGURE 2:**
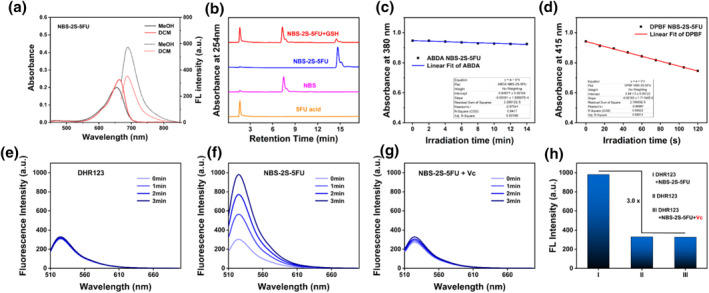
(a) UV‐vis absorbance (solid line, left) and fluorescence spectra (dash line, right) of NBS‐2S‐5FU (3.0 μM) in various solvents. (b) HPLC analysis of 5‐FU acid (10.0 μM), NBS (10.0 μM), and NBS‐2S‐5FU (10.0 μM) with or without GSH (2.5 mM) for 2 h at 37°C. (c) Absorption changes of ABDA at 380 nm. (d) Absorption changes of DPBF at 415 nm. Fluorescence curve of (e) DHR123 (f) DHR123 + NBS‐2S‐5FU (3.0 μM) and (g) DHR123 + NBS‐2S‐5FU (3.0 μM) + Vc (radical scavenger, 0.5 mM) at different times. (h) Fluorescence intensity of DHR123 at 524 nm after 3 min of irradiation (660 nm, 5 mW/cm^2^).

To investigate the photophysical properties of NBS‐2S‐5FU, the UV‐vis absorption and fluorescence spectrum were studied in MeOH and DCM (Figure 2a). NBS‐2S‐5FU displayed an intense absorption profile at 656 nm and could achieve a maximum emission value of approximately 690 nm, which was beneficial for tracking and imaging during the treatment process. Subsequently, the release of NBS and 5‐FU derivatives from GSH‐treated NBS‐2S‐5FU was assessed using HPLC (Figure 2b). NBS‐2S‐5FU showed the peak with a retention time of 14.7 min in the HPLC spectrum. After treatment with GSH, two new peaks appeared at 1.6 and 7.5 min, which was consistent with the standard retention times of 5‐FU derivatives and NBS under the same conditions, respectively. The HPLC results indicated that NBS and 5‐FU derivatives could be released from NBS‐2S‐5FU upon activation of GSH.

### 2.2 Light triggered ROS generation

In recent years, there had been significant advancement in the use of light for PDT, controlled release of active substances, and treatment of bacterial infections.^[13]^ The reactive oxygen species (ROS) generated by light were particularly pivotal in these processes. To confirm the types of ROS produced by NBS‐2S‐5FU under light irradiation, the production of singlet oxygen (^1^O_2_) and $O_{2}^{-•}$ was measured using probes including ABDA, DPBF, and DHR123.^[14]^ The ^1^O_2_ generation was first tested with corresponding detection reagents, and the absorption of ABDA and DPBF hardly decreased after irradiation, indicating NBS‐2S‐5FU showed weak ^1^O_2_ generation ability during the irradiation process (Figure 2c and d and Figure S2). Next, $O_{2}^{-•}$ detection was performed. After irradiated for 3 min, no fluorescence increase was detected at 524 nm when using DHR123 alone (Figure 2e). However, the addition of NBS‐2S‐5FU increased the fluorescence emission of DHR123 by 3 times (Figure 2f). Moreover, vitamin C (Vc),^[15]^ a radical scavenger, was added to the solution, and as expected, the fluorescence intensity at 524nm hardly increased under a 3 min irradiation period (Figures 2g and h). Taken together, the above characterizations suggested that NBS‐2S‐5FU predominantly generated $O_{2}^{-•}$ during the PDT process.

### 2.3 Cell uptake and subcellular accumulation

The enrichment concentration and membrane penetration ability of small molecule drugs in tumor cells are considered to be two crucial factors influencing the therapeutic efficacy. Next, the cellular uptake capacity of NBS‐2S‐5FU was investigated using 4T1 cells on a confocal laser scanning microscope (CLSM). NBS‐2S‐5FU showed efficient cellular uptake after incubation with cells for 30 min and reached its maximum concentration at 90 min (Figure S3). Colocalization studies showed that NBS‐2S‐5FU exhibited a high level of co‐localization with Lyso Tracker Green in both 4T1 and B16 cells, confirming that NBS‐2S‐5FU accumulated preferentially in the lysosome (Figure S4).

### 2.4 Intracellular GSH concentration

Subsequently, the intracellular concentration of GSH was quantified, and as anticipated, the GSH level in cancer cells was approximately 8 times higher than that in normal cells (mouse fibroblast cells, 3T3), meeting the criteria for prodrug release (Figure S5).

### 2.5 Photoinduced ROS generation in cells

**FIGURE 3:**
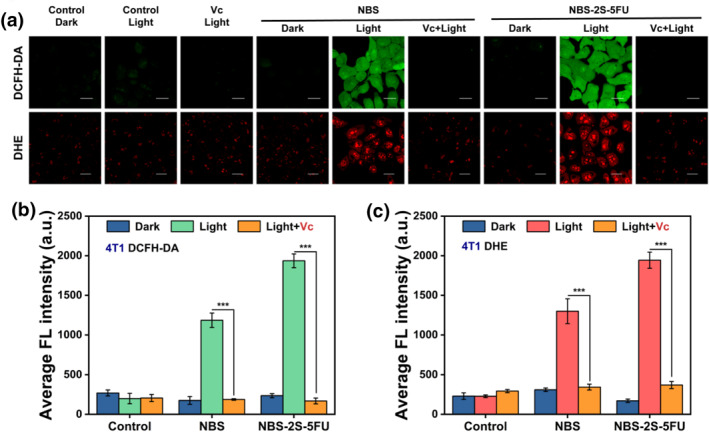
(a) Intracellular reactive oxygen species and $O_{2}^{-•}$ generation of 4T1 cells after various treatments. DCF (green channel): *λ*_ex_ = 488 nm, *λ*_em_ = 500–550 nm. Vc (0.5 mM) was used as the $O_{2}^{-•}$ scavenger. DHE (10 μM, red channel): *λ*_ex_ = 488 nm, *λ*_em_ = 570–630 nm. Scale bars, 20 μm. (b) Average fluorescence (FL) intensity of green fluorescence of DCF in 4T1 cells. (c) Average FL intensity of red fluorescence of DHE in 4T1 cells. Data are shown as mean ± s.d. (statistical significances were calculated using Student's *t*‐test: ***p < 0.001).

Then, the ability of NBS‐2S‐5FU to induce ROS generation in 4T1 cells was investigated (Figure 3a and b). DCFH‐DA was used as an indicator of the intracellular ROS.^[16]^ It could be noted that the ROS generation was obvious in the NBS‐2S‐5FU and NBS light groups, which showed apparent green fluorescence from oxidized DCF. The photoinduced intracellular $O_{2}^{-•}$ generation was investigated using DHE as the fluorescent probe.^[17]^ Confocal imaging disclosed that NBS‐2S‐5FU and NBS could generate $O_{2}^{-•}$ efficiently in 4T1 cells upon irradiation. Remarkably, 4T1 cells pretreated with Vc before staining photosensitizers exhibited a rapid decrease in fluorescence intensity due to the ROS and $O_{2}^{-•}$ scavenging properties of Vc (Figure 3c). Also, a similar phenomenon was reflected in B16 cells (Figure S6). These results provided strong evidence that NBS‐2S‐5FU had the potential to be effective in PDT due to its ability to efficiently generate $O_{2}^{-•}$ against tumor cells.

### 2.6 In vitro cytotoxic study

**FIGURE 4:**
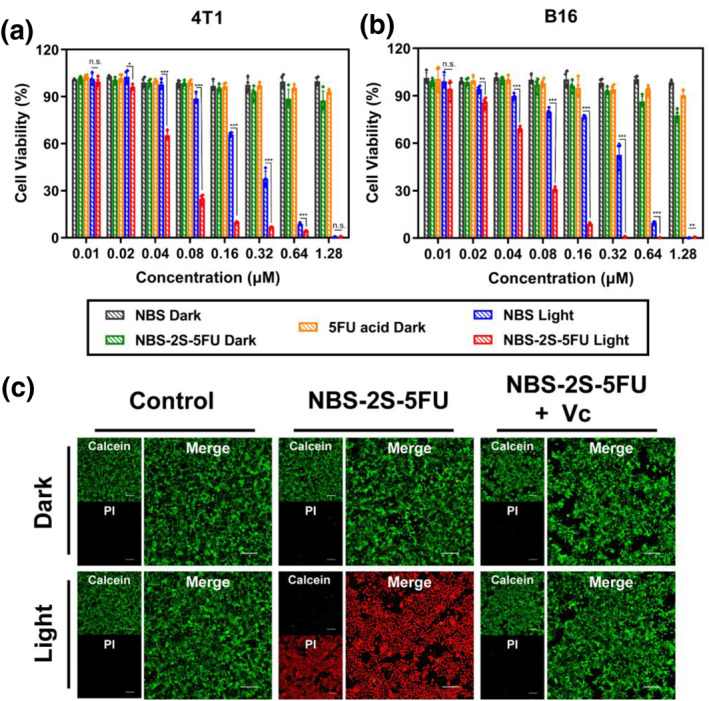
Cell viability of (a) 4T1 and (b) B16 cells with 5‐FU acid, NBS, and NBS‐2S‐5FU in the presence or the absence of light. *N* = 4. (c) Imaging of Calcein‐AM (green channel, living cells, *λ*_ex_ = 488 nm, *λ*_em_ = 500–550 nm) and PI (red channel, dead cells, *λ*_ex_ = 561 nm, *λ*_em_ = 580–630 nm) staining of 4T1 cells after different treatments. The concentrations of NBS‐2S‐5FU and Vc are 0.5 μM and 0.5 mM, respectively. Scale bars, 200 μm. Data are shown as mean ± s.d. (statistical significances were calculated by Student's *t*‐test: *p < 0.05, **p < 0.01, ***p < 0.001).

The therapeutic efficacy of NBS‐2S‐5FU was assessed by studying the cytotoxicity using MTT assays on 5 different types of cells (Figure 4a and b and Figure S7‐S8).^[18]^ The cell viability of all drugs on tumor cells (4T1, B16, MCF‐7, Hela) and normal cells (3T3) remained above 80% without light irradiation, indicating their favorable biocompatibility in vitro. Strong cell‐killing ability was exhibited with the half inhibitory concentration (IC_50_) being low to 52 nM in 4T1 cells and 57 nM in B16 cells, indicating the combination of PDT and 5‐FU derivatives was efficiently performed. Moreover, Calcein‐AM (green, living cells) and PI (red, dead cells) were used to visually monitor cell viability.^[19]^ Clearly, nearly all cells treated with NBS‐2S‐5FU showed strong red fluorescence with negligible green fluorescence after irradiation. Upon comparison, the other groups exhibited very weak red fluorescence and strong green fluorescence, indicating the predominance of live cells (Figure 4c). The potent cytotoxic effect of NBS‐2S‐5FU was also observed in other cancer cell lines under the same experimental conditions (Figure S9).

### 2.7 Apoptosis induced by NBS‐2S‐5FU

**FIGURE 5:**
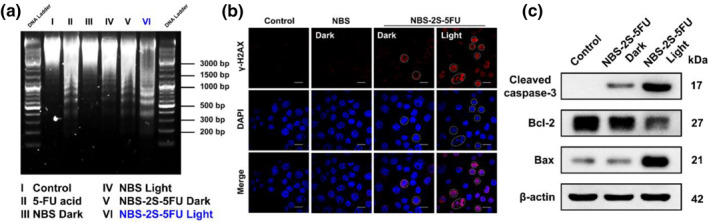
(a) Agarose gel electrophoresis in 4T1 cells with different treatments. (b) *γ*‐H2AX immunofluorescence in 4T1 cells with different treatments, including NBS (500 nM), NBS‐2S‐5FU (500 nM), and NBS‐2S‐5FU (500 nM) + light. Scale bars, 20 μm. (c) Western blot analysis of 4T1 cells treated with NBS‐2S‐5FU.

To get a better understanding of the cooperative therapeutic mechanism for NBS‐2S‐5FU, further cellular apoptosis studies were explored in 4T1 cells. 5‐FU and its derivatives are pyrimidine antimetabolic drugs that can inhibit DNA biosynthesis and induce cell apoptosis.^[20]^ When cells undergo apoptosis, endonucleases are activated and cleave DNA, causing DNA to break and form oligonucleotide fragments. Based on this feature, DNA fragmentation ladders were detected by agarose gel electrophoresis in the first place. As clearly depicted in Figure 5a, the purified DNA in NBS‐2S‐5FU + light groups exhibited the most typical “ladder‐like” apoptotic characteristics, with a higher degree of cell apoptosis than other groups. In addition, H2AX phosphorylated at serine 139 (γ‐H2AX) is a specific indicator for the existence of a DNA double‐strand break.^[21]^ Therefore, the *γ*‐H2AX immunofluorescence was performed next. As depicted in Figure 5b, following incubation with NBS‐2S‐5FU, cells in the dark group exhibited partial red *γ*‐H2AX fluorescence within the nucleus, which was subsequently significantly enhanced upon PDT treatment, indicating the induction of DNA damage and promotion of apoptosis by PDT. However, no significant DNA damage signals were detected in either the NBS or control groups. Next, Annexin V‐FITC/PI double staining was performed to verify the apoptosis in tumor cells.^[22]^ As shown in Figure S10, the green fluorescence of FITC and the red fluorescence of PI were only observed simultaneously in the PDT group, which means the occurrence of apoptosis progress. In contrast, negligible apoptosis signals were observed in other groups. Finally, to confirm the overall apoptosis of cells, various apoptosis indicators were assessed through western blot experiments. As clearly depicted in Figure 5c, NBS‐2S‐5FU treatment resulted in a decrease in Bcl‐2 (an anti‐apoptotic protein) expression, an increase in BAX (a pro‐apoptotic protein) expression, and a significant elevation of activated caspase‐3 expression upon light irradiation, indicating the induction of extensive cellular apoptosis.^[23]^ When NBS‐2S‐5FU was used without irradiation, there was a slight upregulation in the expression of activated caspase‐3, and only a slight level of apoptosis was observed. Collectively, these observations suggested that drugs released by NBS‐2S‐5FU could inhibit DNA biosynthesis and exert a synergistic effect with PDT, ultimately leading to cancer cell apoptosis.

### 2.8 In vivo antitumor evaluation

**FIGURE 6:**
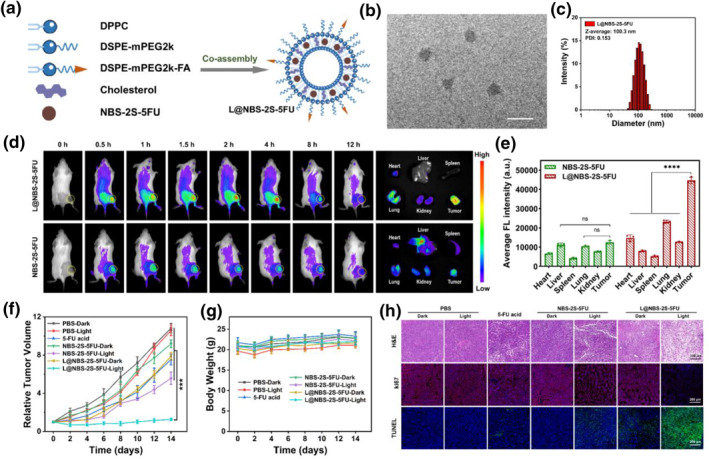
(a) Diagram of preparation of liposomes L@NBS‐2S‐5FU (b) transmission electron microscopy characterization of L@NBS‐2S‐5FU. Scale bars, 200 nm. (c) dynamic light scattering measurement of L@NBS‐2S‐5FU. (d) In vivo fluorescence imaging of Balb/c mice after *i*.*v*. injection of L@NBS‐2S‐5FU or NBS‐2S‐5FU (100 μL, 0.2 mg/kg). *λ*_ex_ = 630 nm, *λ*_em_ = 680–720 nm. Fluorescence imaging of major organs and tumor at 4 h after *i*.*v*. injection of drugs. (e) Average fluorescence (FL) intensity of major organs and tumor at 4 h after *i*.*v*. injection of drugs. (f) Changes in the tumor volume of the 4T1 tumor model. (g) Changes in the body weight of mice following the administration of treatments. (h) H&E, ki67, and TUNEL staining of tumor slides from mice after different treatments. Scale bars, 200 μm. Data are shown as mean ± s.d. (statistical significances were calculated by Student's *t*‐test: ***p < 0.001, ****p < 0.0001).

Due to the outstanding therapeutic efficacy of NBS‐2S‐5FU in vitro, Balb/c mice were employed for investigating the in vivo anti‐tumor activity of NBS‐2S‐5FU. Firstly, the presence of a significant amount of GSH (≤1.02 mM) and albumin protein with vicinal cysteine residues in blood serum might lead to the premature release of chemotherapeutic agents.^[24]^ Furthermore, in comparison to the direct utilization of drug monomers, the use of liposomes for drug delivery was associated with a heightened efficiency in tumor‐specific drug enrichment. With these characteristics in mind, a liposome‐based delivery system was utilized for the loading of NBS‐2S‐5FU. The NBS‐2S‐5FU‐loaded liposome (L@NBS‐2S‐5FU) was successfully prepared referring to our previous study (Figure 6a).^[25]^ Therein, liposome formulation utilized synthetic phospholipids of DSPE‐mPEG2k‐FA, which not only enhanced active targeting through the overexpression of folate receptors in tumors but also effectively evaded recognition and phagocytosis by the reticuloendothelial system (RES) due to the presence of a PEG framework.^[26]^ The encapsulation efficiency of L@NBS‐2S‐5FU was 77%, and the drug loading rate was 1.15%. The transmission electron microscopy (TEM) revealed that the average diameter of L@NBS‐2S‐5FU was approximately 90 nm, exhibiting a spherical structure with uniform size and regular morphology (Figure 6b). In an aqueous solution, the L@NBS‐2S‐5FU hydrated particle size exhibited a normal and unimodal distribution as determined by dynamic light scattering (DLS) (Figure 6c).

Subsequently, to monitor the tumor accumulation behavior and organ distribution of drugs, 4T1 tumor‐bearing mice were treated with NBS‐2S‐5FU and L@NBS‐2S‐5FU via intravenous *(i*.*v*.) injection, respectively. Meanwhile, the fluorescence imaging was observed at different post‐injection times. As shown in Figure 6d and Figure S11, a significant accumulation of L@NBS‐2S‐5FU at the tumor site after 4 h *i*.*v*. injection of liposome drugs, while the NBS‐2S‐5FU itself barely reached the tumor site after *i*.*v*. injection. The mice were sacrificed and dissected after 4 h *i*.*v*. injection, revealing that L@NBS‐2S‐5FU primarily accumulated in the tumor, and NBS‐2S‐5FU accumulated slightly at the tumor site (Figure 6e).

By leveraging its targeting ability and high collaborative treatment efficiency, L@NBS‐2S‐5FU demonstrated a satisfactory anti‐tumor efficacy in vivo. As shown in Figure 6f, invasive tumor growth was observed in the PBS, 5‐FU acid, or NBS‐2S‐5FU treatment group, whereas L@NBS‐2S‐5FU with PDT efficiently suppresses the tumor growth. The outstanding antitumor performance of L@NBS‐2S‐5FU was further demonstrated by the representative tumor images (Figure S12‐S13) as well as H&E staining of tumor slices (Figure 6h). Meanwhile, no significant weight loss was observed throughout the entire treatment process (Figure 6g) and no obvious physiological damage or inflammatory lesions in the H&E staining of major organs (Figure S14), suggesting all drugs were biologically safe in vivo. Ki67 is an antigen closely associated with cell proliferation, playing an essential role in the mitotic process of cell division. It serves as a reliable indicator of tumor cell proliferation activity. Furthermore, TUNEL tests are capable of detecting DNA ends that have been broken during apoptosis. As shown in Figure 6h, ki67 staining of tumor sections intuitively displayed the least red fluorescent proliferative cells in L@NBS‐2S‐5FU with PDT compared with others, suggesting repressive proliferation induced by L@NBS‐2S‐5FU with irradiation. TUNEL assays witnessed the exceptional antitumor efficiency of L@NBS‐2S‐5FU with PDT in vivo with conspicuous green fluorescence signals on behalf of the intense apoptosis.

## 3 CONCLUSION

In conclusion, focusing on GSH overexpression in the tumor microenvironment, a GSH‐responsive intelligent activation prodrug is designed. After the prodrug is activated, it releases the photosensitizer NBS and the chemotherapeutic agent 5‐FU derivatives. Notably, low doses of light exposure effectively suppress tumor growth in vitro. Moreover, the synergistic effect of combining PDT with 5‐FU derivatives not only effectively inhibits DNA biosynthesis but also amplifies the photocytotoxicity of PDT, culminating in the induction of cell apoptosis. Furthermore, in vivo studies have demonstrated that L@NBS‐2S‐5FU exhibits a robust tumor suppression effect following intravenous injection. This innovative approach has the potential to minimize the use of chemotherapy and photosensitizer drugs, ensuring treatment effectiveness simultaneously. Consequently, this pathway presents an attractive prospect for advancing combined prodrugs that integrate PDT and chemotherapy.

## CONFLICT OF INTEREST STATEMENT

The authors declare no conflicts of interest.

## ETHICS STATEMENT

All animal experiments were performed according to the guidelines of the Care and Use of Laboratory Animals formulated by the Ministry of Science and Technology of China and were approved by the Animal Care and Use Committee of Dalian University of Technology (2019‐018).

## Supporting information

Supporting Information S1

## Data Availability

The data that support the findings of this study are available from the corresponding author upon reasonable request.
