# Supplementary material for: A self‐reinforced activatable photosensitizer prodrug enabling synergistic photodynamic and chemotherapy
Source: Smart Mol. 2024 Jun 14;2(4):e20240005. doi: 10.1002/smo.20240005 (PMC12118193; doi:10.1002/smo.20240005)
Supplement: Supplementary file 1 — Supporting Information S1 [file SMO2-2-e20240005-s001.pdf]

Supporting Information

A Self-reinforced Activatable Photosensitizer Prodrug Enabling Synergistic Photodynamic and Chemotherapy

*Daipeng Huang, Jikai Yin, Yang Zou, Haiqiao Huang, Saran Long, Wen Sun, Jianjun Du, Jiangli Fan, Xiaojun Peng**

D. Huang, J. Yin, Y. Zou, H. Huang, S. Long, W. Sun, J. Du, J. Fan, X. Peng.
State Key Laboratory of Fine Chemicals, Frontiers Science Center for Smart Materials Oriented Chemical Engineering, Dalian University of Technology, Dalian 116024, P. R. China.
E-mail: pengxj@dlut.edu.cn

H. Huang, X. Peng.
State Key Laboratory of Fine Chemicals, College of Materials Science and Engineering, Shenzhen University, Shenzhen, 518071, P. R. China.

Y. Zou.
Key Laboratory of Biotechnology and Bioresources Utilization of Ministry of Education, College of Life Science, Dalian Minzu University, Dalian 116600, P. R. China.

Content

1 Supporting figures 3
2 Experimental section 9
- 2.1 Materials and instrumentation 9
- 2.2 Synthesis of products 10
- 2.3 High-performance liquid chromatography 11
- 2.4 Singlet oxygen ($^1O_2$) detection 11
- 2.5 Superoxide anion radical ($O_2^{-•}$) detection 11
- 2.6 Cell culture 12
- 2.7 Cell uptake real-time imaging 12
- 2.8 *In vitro* GSH assays 12
- 2.9 Subcellular colocalization assay 12
- 2.10 Intracellular ROS detection 13
- 2.11 Intracellular superoxide anion radical ($O_2^{-•}$) detection 13
- 2.12 Cell viability assay (MTT assay) 13
- 2.13 Dead/live cell co-staining 13
- 2.14 Agarose gel electrophoresis assay 14
- 2.15 γ-H2AX immunofluorescence assay 14
- 2.16 Annexin V-FITC/PI co-staining assay 14
- 2.17 Western blot assay 14
- 2.18 Preparation of L@NBS-2S-5FU 15
- 2.19 *In vivo* antitumor evaluation 15

3 HRMS, ^1H and ^{13}C NMR spectra 17
4 References 18

1 Supporting figures

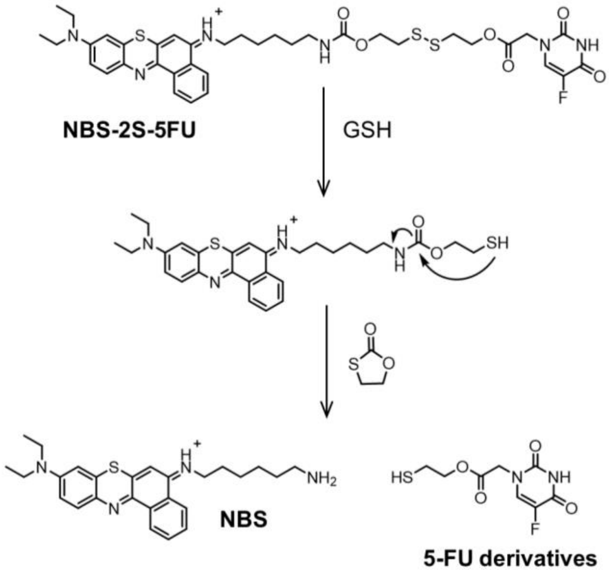

Figure S1. Proposed NBS and drug release mechanism of the activable **NBS-2S-5FU** by GSH.

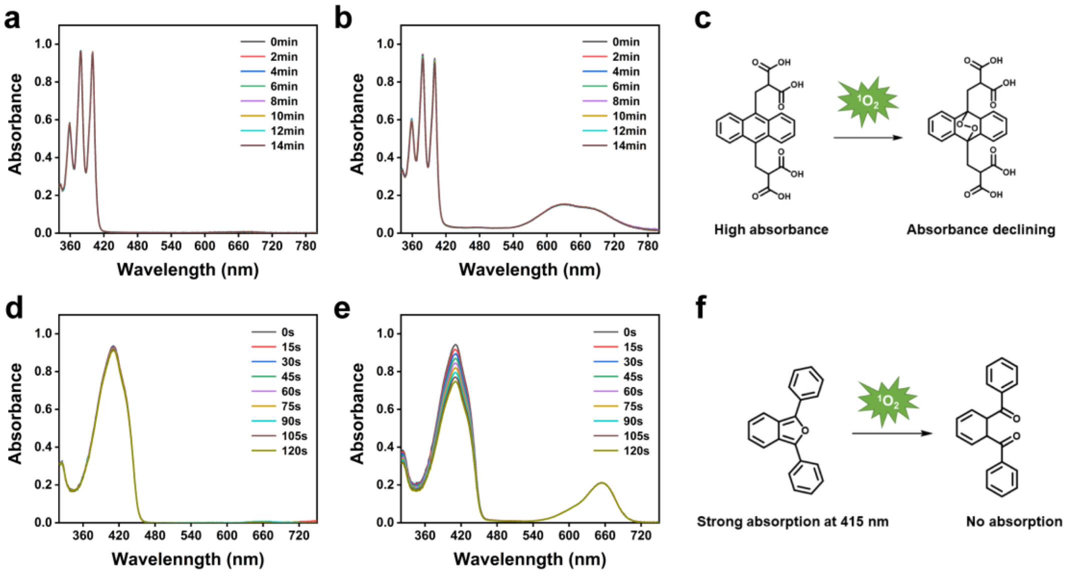

Figure S2. (a) Absorption curves of ABDA at different times. (b) Absorption curves of ABDA and **NBS-2S-5FU** (3 μM) at different times. (c) Schematic illustration of ABDA for detecting $^1O_2$ generation. (d) Absorption curves of DPBF at different times. (e) Absorption curves of DPBF and **NBS-2S-5FU** (3 μM) at different times. (f) Schematic illustration of DPBF for detecting $^1O_2$ generation.

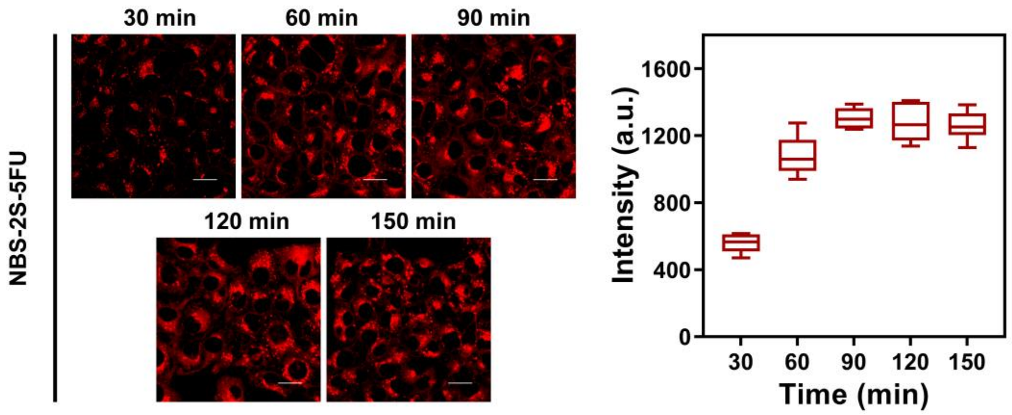

Figure S3. Cellular uptake of **NBS-2S-5FU** in 4T1 cells. NBS-2S-5FU (500 nM, red channel): λ_{ex} = 640 nm, λ_{em} = 645-700 nm. Scale bars, 20 µm.

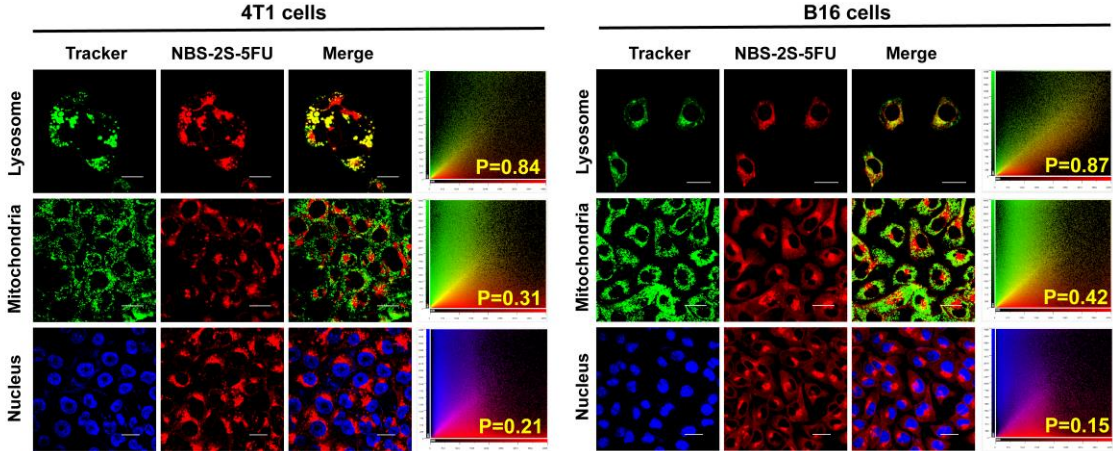

Figure S4. 4T1 and B16 cells subcellular colocalization imaging. Mito and Lyso Tracker (100 nM, green channel): λ_{ex} = 488 nm, λ_{em} = 500-550 nm. Nucleus Tracker (100 nM, blue channel): λ_{ex} = 405 nm, λ_{em} = 440-480 nm. **NBS-2S-5FU** (500 nM, red channel): λ_{ex} = 640 nm, λ_{em} = 645-700 nm. Scale bars, 20 µm.

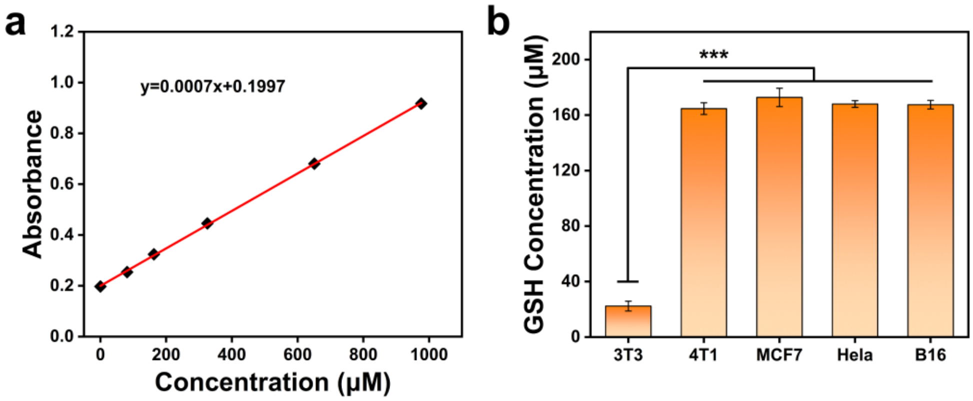

Figure S5. (a) Standard curve of GSH content. (b) GSH concentration of different kinds of cells.

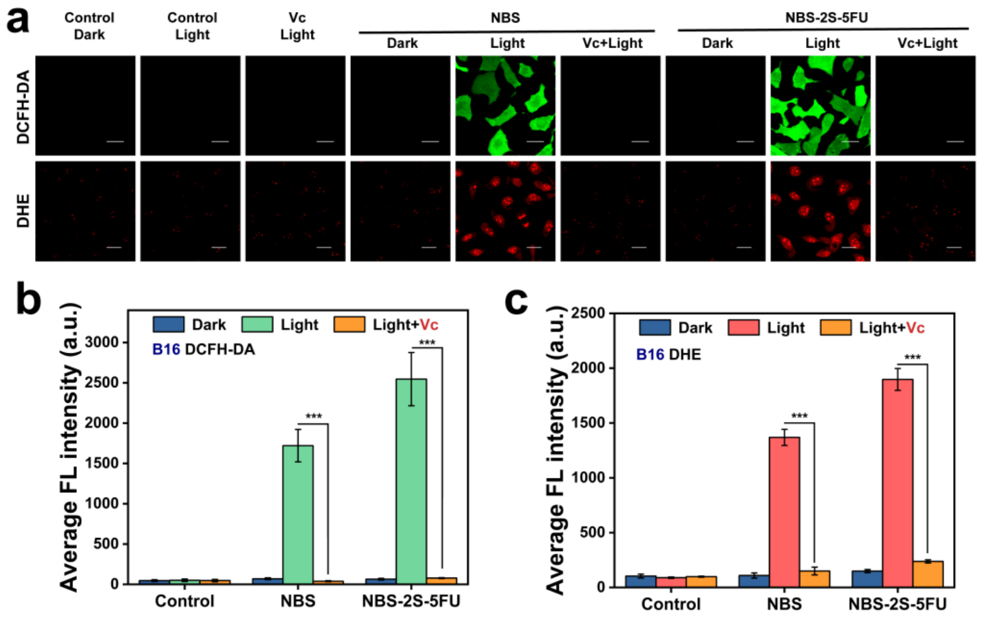

Figure S6. (a) Intracellular ROS and $O_2^{-\bullet}$ generation of B16 cells after various treatments. DCF (green channel): λ_{ex} = 488 nm, λ_{em} = 500-550 nm. Vc (0.5 mM) was used as the $O_2^{-\bullet}$ scavenger. DHE (10 μM, red channel): λ_{ex} = 488 nm, λ_{em} = 570-630 nm. Scale bars, 20 μm. (b) Average fluorescence (FL) intensity of green fluorescence of DCF in B16 cells. (c) Average FL intensity of red fluorescence of DHE in B16 cells.

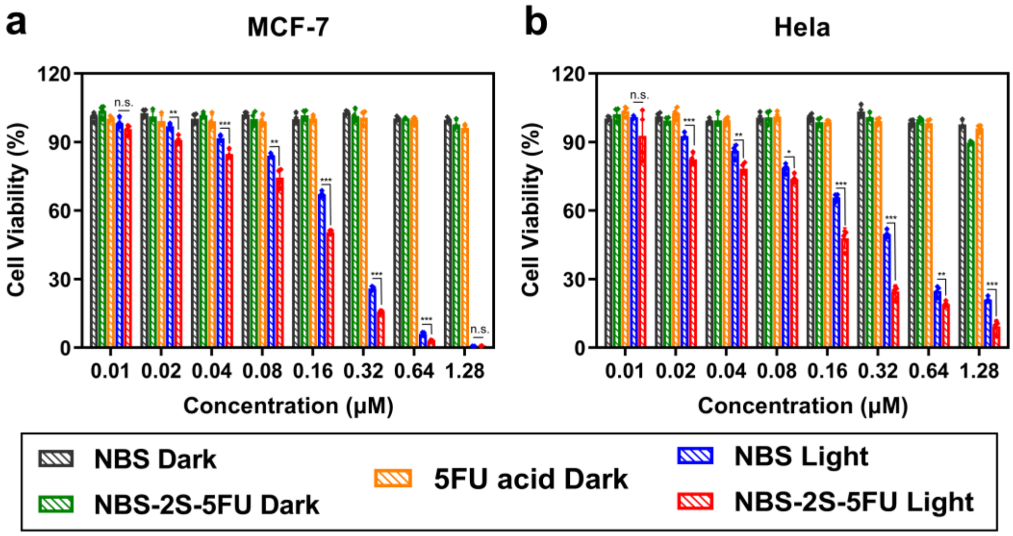

Figure S7. Cell viability of (a) MCF-7 and (b) Hela cells with 5-FU acid, NBS, and **NBS-2S-5FU** in the presence or the absence of light. N = 4. Data are shown as mean ± s.d. (statistical significances were calculated by Student's t-test: *p < 0.05, **p < 0.01, ***p < 0.001).

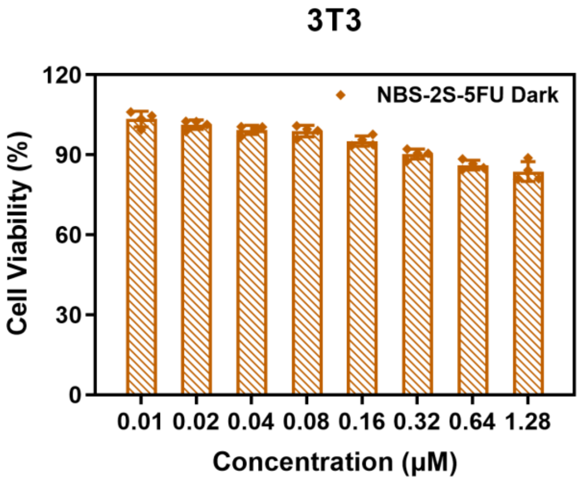

Figure S8. Cell viability of 3T3 cells treated with **NBS-2S-5FU** in the absence of light. N = 4.

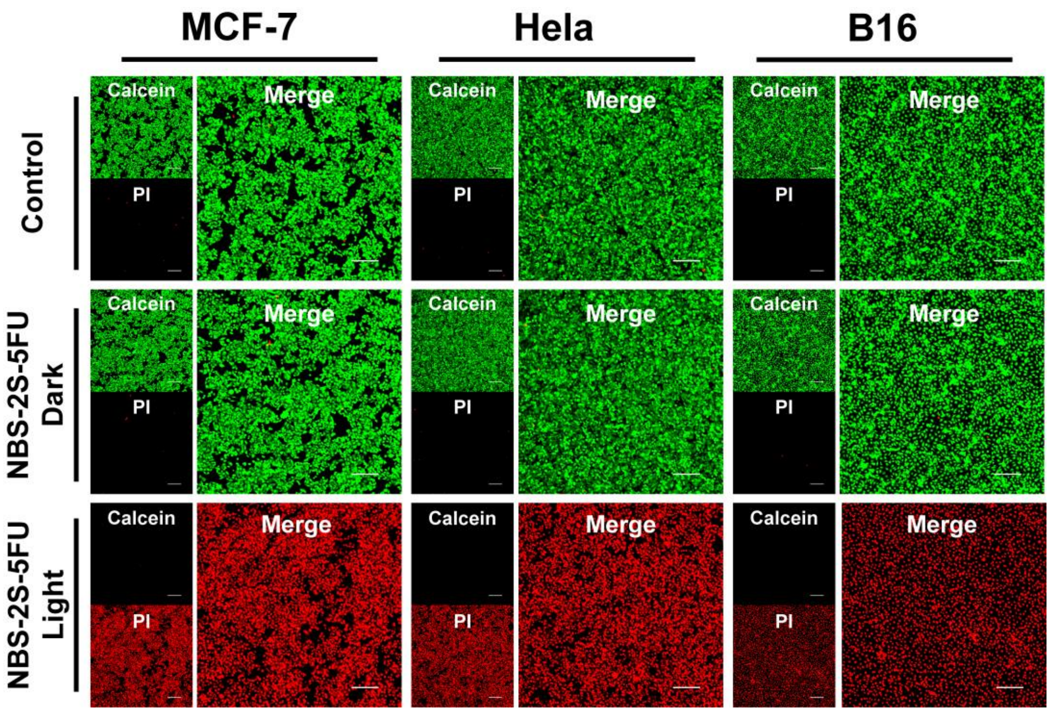

Figure S9. Imaging of Calcein-AM (green channel, living cells, λ_{ex} = 488 nm, λ_{em} = 500-550 nm) and PI (red channel, dead cells, λ_{ex} = 561 nm, λ_{em} = 580-630 nm) staining of MCF-7, Hela, and B16 cells after different treatments. Scale bars, 200 μm.

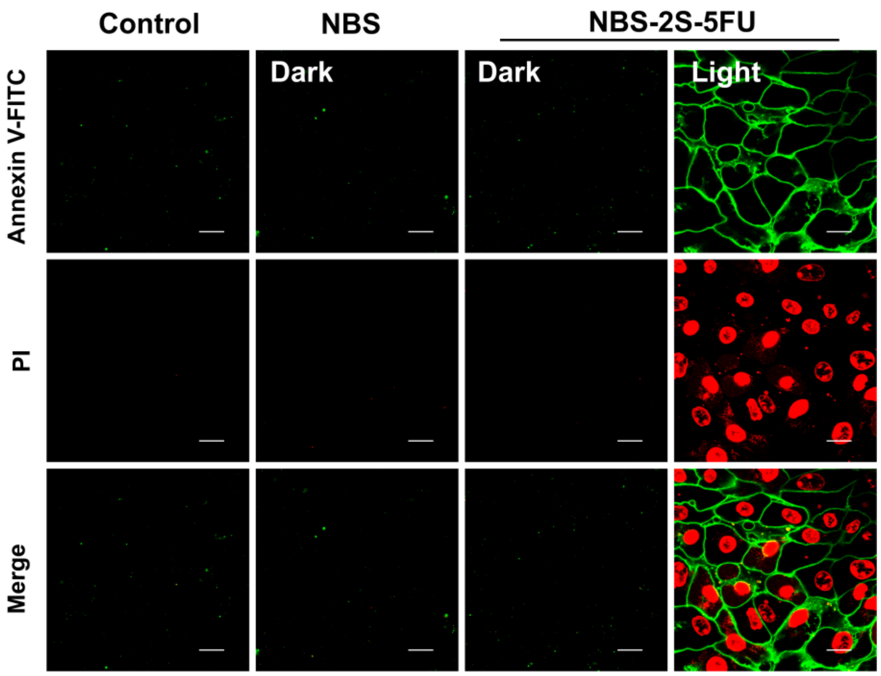

Figure S10. Annexin V-FITC/PI co-staining on 4T1 cells after different treatments. Annexin V-FITC (green channel, λ_{ex} = 488 nm, λ_{em} = 500-550 nm). PI (red channel, λ_{ex} = 488 nm, λ_{em} = 600-700 nm). Scale bars, 20 μm.

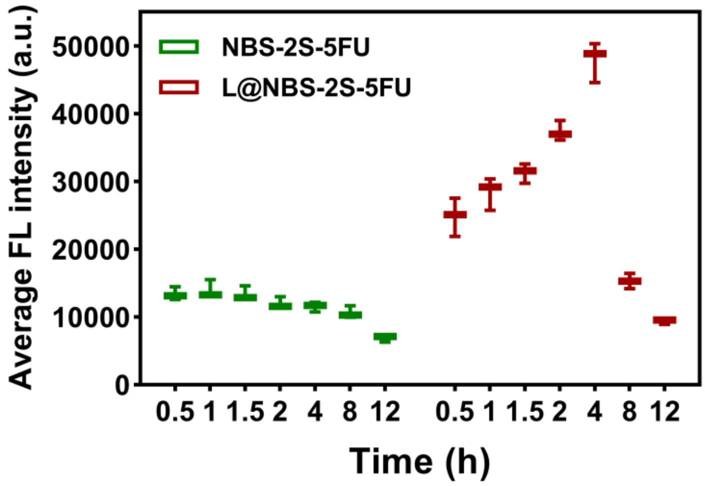

Figure S11. Semi-quantitative analysis of fluorescence at tumor sites in mice.

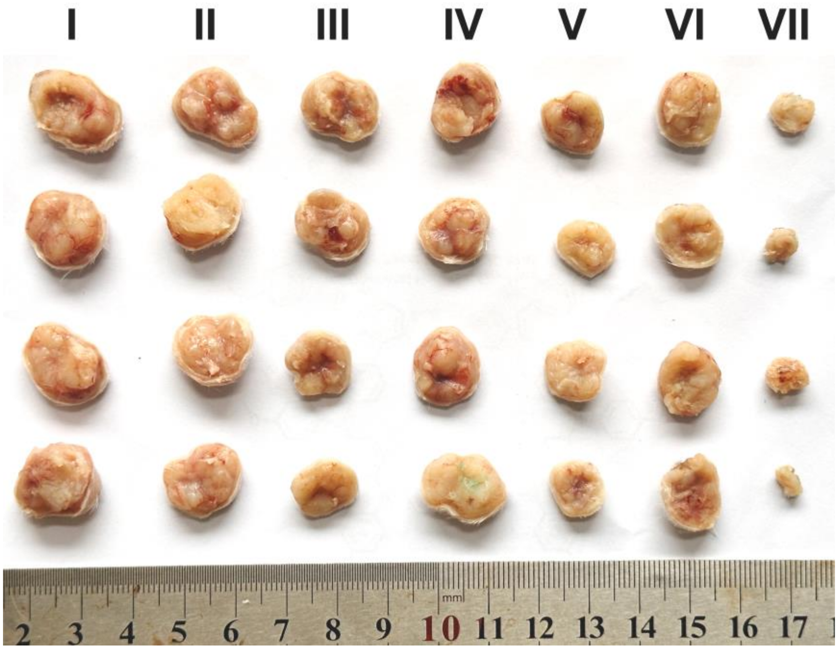

Figure S12. Photo of tumors after different treatments. (I) PBS Dark, (II) PBS Light, (III) 5-FU acid, (IV) **NBS-2S-5FU** Dark, (V) **NBS-2S-5FU** Light, (VI) **L@NBS-2S-5FU** Dark, (VII) **L@NBS-2S-5FU** Light.

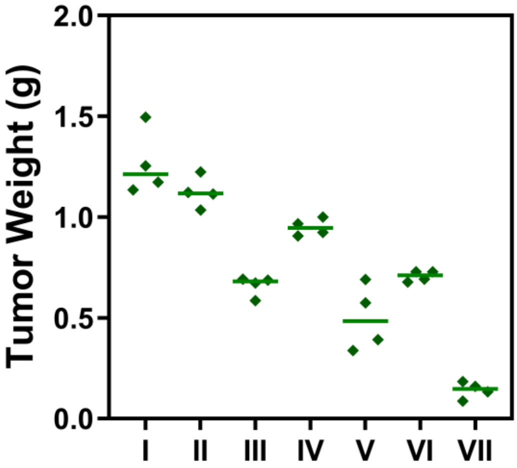

Figure S13. Tumor weight in different groups at 14th day post-injection. I) PBS Dark, II) PBS Light, III) 5-FU acid, IV) **NBS-2S-5FU** Dark, V) **NBS-2S-5FU** Light, VI) **L@NBS-2S-5FU** Dark, VII) **L@NBS-2S-5FU** Light. N=4.

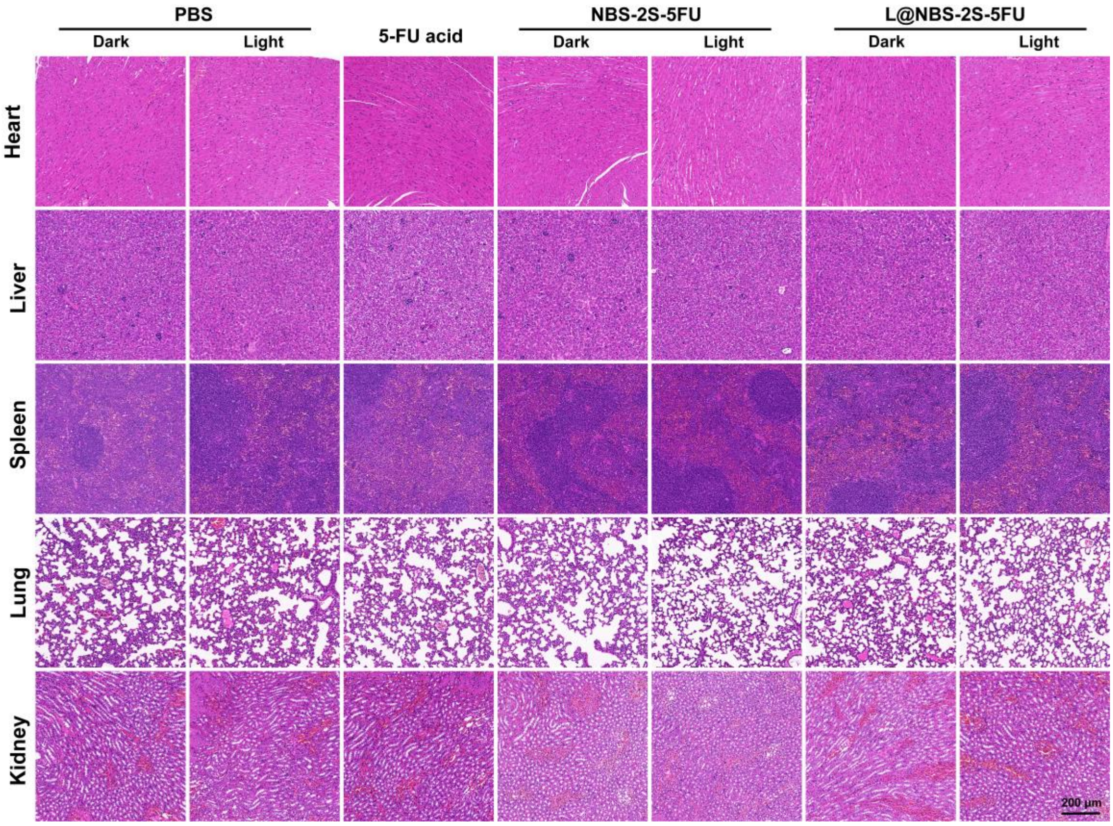

Figure S14. Images of H&E staining of major organs slides from mice after different treatments. Scale bars, 200 μm.

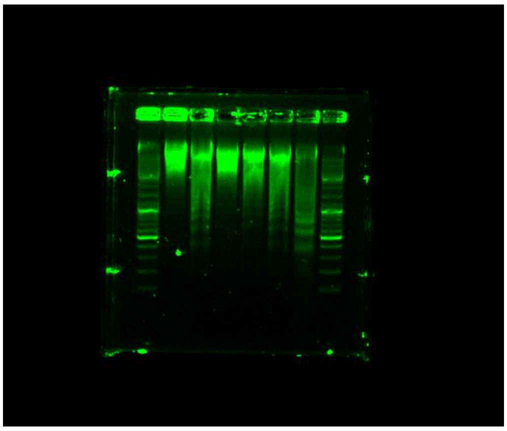

Figure S15. Uncropped agarose gel electrophoresis source data for Figure 5a.

2 Experimental section

2.1 Materials and instrumentation

The general chemicals including 3-(4,5-dimethylthiazol-2-yl)-2,5-diphenyltetrazolium bromide (MTT), 9,10-anthracenediyl-bis(methylene)-dimalonic acid (ABDA), GSH, Vc, 5-FU, HATU, DIPEA, bromoacetic acid, and 1,3-diphenylisobenzofuran (DPBF) were purchased from Energy Chemical Co. (Shanghai, P. R. China). Dihydroethidium (DHE), DCFH-DA, Hoechst 33342,

Calcein/PI Cell Viability/Cytotoxicity Assay Kit, Annexin V-FITC/propidium iodide (PI) Detection Kit, DNA Damage Assay Kit by γ-H2AX Immunofluorescence, Universal Genomic DNA Purification Mini Spin Kit, Bcl-2 Rabbit Polyclonal Antibody, BAX Rabbit Polyclonal Antibody, and β-Actin Mouse Monoclonal Antibody were purchased from Beyotime Biotechnology Co., Ltd. (Shanghai, P. R. China). Reduced Glutathione (GSH) Content Assay Kit and DNA Loading Buffer (6×) were purchased from Solarbio Science & Technology Co., Ltd. (Beijing, P. R. China). LysoTracker Green DND 26 and MitoTracker Green FM were purchased from Thermo Fisher Scientific Inc. (Shanghai, P. R. China). Milli-Q water was supplied by Milli-Q Plus System (Millipore Corporation, U.S.A). Cleaved Caspase-3 Antibody (catalogue #9664) was purchased from Cell Signaling Technology (U.S.A). HRP-conjugated Affinipure Goat Anti-Mouse IgG(H+L) (catalogue #SA00001-1) and HRP-conjugated Affinipure Goat Anti-Rabbit IgG(H+L) (catalogue #SA00001-2) were purchased from Proteintech (Wuhan, P. R. China).

All the other solvents and reagents used in this study were of analytical grade.

Mouse fibroblast cells (3T3), Mouse breast cancer cells (4T1), Human breast cancer cells (MCF7), Human cervical cancer cells (Hela), and Mouse melanoma cells (B16) were purchased from the Cell Resource Center, Peking Union Medical College (PCRC).

^{1}H NMR and ^{13}C NMR spectra were detected by a Bruker Avance NEO 600M NMR Spectroscopy (Bruker, Switzerland). Mass spectrometry (MS) was carried out using ion mobility quadrupole time-of-flight tandem mass spectrometer (Waters, Synapt G2-Si HDMS, U.S.A). Absorption spectra were measured on a CARY 60 UV-Vis spectrophotometer (Agilent, U.S.A). Fluorescence spectra were obtained with an Agilent Cary Eclipse fluorescence spectrophotometer (Agilent, U.S.A). Confocal laser scanning microscope (CLSM) images were performed on an Olympus FV3000 CLSM (Olympus, Japan). Small animals' fluorescence imaging was carried out by the NightOWL II LB983 living imaging system (Berthold Technologies, Germany).

2.2 Synthesis of products

NBS-2S-OH was synthesized according to our previous method.[1]

5-FU acid was synthesized according to the literature method. [2,3]

Synthesis of ***NBS-2S-5FU***

5-FU acid (235 mg, 1.25 mmol), HATU (475 mg, 1.25 mmol) and DIPEA (350 μL, 2 mmol) were dissolved in DMF (6 mL), after stirring at room temperature for 30 minutes, NBS-2S-OH (613 mg, 1 mmol) dissolved in DMF (6 mL) and dropped into the above mixed system. The mixture was stirred for 12 h before the addition of water (30 mL). The aqueous mixture was extracted three times with ethyl acetate (10 mL each time) and the organic phase was washed twice with brine (10 mL each time), dried over anhydrous sodium sulfate, and the organic phase was evaporated under reduced pressure. The resulting crude product was purified via silica gel column chromatography ($CH2Cl_2$-methanol = 10:1, v/v) to yield **NBS-2S-5FU** as a dark blue solid (324 mg, 41.4% yield). ^{1}H NMR (600 MHz, DMSO) δ 11.98 (s, 1H), 9.75 (s, 1H), 9.01 (d, 1H), 8.51 (d, 1H), 8.32 (s, 1H), 8.08-7.86 (m, 4H), 7.56 (s, 1H), 7.42 (s, 1H), 7.20 (t, 1H), 4.49 (s, 2H), 4.34 (t, 2H), 4.16 (t, 2H),

3.68 (dt, 6H), 2.96 (dt, 6H), 1.79-1.73 (m, 2H), 1.46-1.40 (m, 4H), 1.37-1.32 (m, 2H), 1.24 (t, 6H). ^{13}C NMR (151 MHz, DMSO) δ 168.25, 157.96, 157.79, 156.38, 153.55, 151.36, 150.06, 140.62, 139.10, 137.38, 134.18, 133.76, 132.65, 131.75, 130.83, 130.60, 130.09, 125.27, 123.67, 117.92, 105.90, 103.57, 79.65, 63.45, 61.95, 49.01, 45.60, 44.55, 37.60, 36.53, 29.77, 28.78, 26.59, 26.41, 13.13 ppm. HRMS (ESI): *m/z* calcd. for $[M]^+$ = 783.2463, found 783.2469.

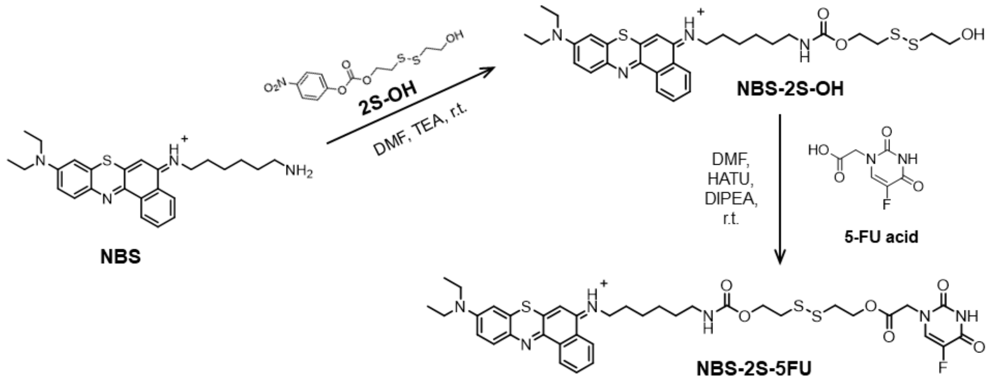

Scheme S1. Synthetic route of **NBS-2S-5FU**.

2.3 High-performance liquid chromatography

For HPLC analysis, deionized water (0.1% formic acid) was used as eluent A and methanol as eluent B. HPLC conditions: 60% of B at 0 min, 80% of B at 15 min, 100% of B at 20 min, 100% of B at 25 min. The injection volume was 20 μL.

The retention time at 1.6 min corresponds to 5-FU acid, 7.5 min corresponds to NBS and 14.7 min corresponds to **NBS-2S-5FU**. The signals were monitored at 254 nm.

2.4 Singlet oxygen ($^1O_2$) detection

The singlet oxygen generated by **NBS-2S-5FU** was measured using 9,10-anthracenediylbis(methylene) dimalonic acid (ABDA) and 1,3-diphenylisobenzofuran (DPBF). Briefly, the absorbance of ABDA at 380 nm was adjusted to about 1.0 in aqueous solution. Then, 3 μM **NBS-2S-5FU** was added to this cuvette. Next, the cuvette was irradiated with 660 nm monochromatic light (power density: 5 mW/cm^2) for various time, and absorption spectra was measured immediately. For DPBF assay, the absorbance of DPBF at 415 nm was adjusted to about 1.0 in MeOH. 3 μM **NBS-2S-5FU** was added to this cuvette. Then the cuvette was exposed to 660 nm monochromatic light (power density: 5 mW/cm^2), and the absorption spectra was observed immediately after each irradiation.

2.5 Superoxide anion radical ($O_2^{-\bullet}$) detection

Dihydrorhodamine 123 (DHR123) was used as the superoxide anion radical indicator. This indicator is converted to Rhodamine 123 in the presence of $O_2^{-\bullet}$. **NBS-2S-5FU** were prepared as 3 μM and DHR123 as 10 μM in aqueous solutions, respectively. When conducting $O_2^{-\bullet}$ quenching experiments, **NBS-2S-5FU** were prepared as 3 μM, DHR123 as 10 μM, and vitamin C (Vc) as 0.5

mM in aqueous solutions, respectively. Cuvettes containing the test article were exposed to 660 nm monochromatic light (power density: 5 mW/cm^2) for different times (1, 2, and 3 min). The fluorescence change of sample was recorded immediately by the fluorescence spectrometer.

2.6 Cell culture

Dulbecco's Modified Eagle Medium (DMEM), Roswell Park Memorial Institute (RPMI) 1640 medium, and phosphate-buffer saline (PBS) buffer with pH = 7.4 are commercial product of Solarbio Science & Technology Co., Ltd (Beijing, P. R. China). Penicillin-Streptomycin Solution (Gibco, #15140122) was purchased from Thermo Fisher Scientific Inc. and Fetal Bovine Serum (FBS) was purchased from PAN Biotech.

Mouse fibroblast cells (3T3), Mouse breast cancer cells (4T1), Human breast cancer cells (MCF7), Human cervical cancer cells (Hela) were cultured in DMEM medium, and Mouse melanoma cells (B16) were cultured in RPMI 1640 medium. All of them were supplemented with 1% penicillin streptomycin and 10% FBS at 37 °C in a CO_2 incubator (5% CO_2).

2.7 Cell uptake real-time imaging

To clarify the cell uptake behaviors of **NBS-2S-5FU**, 4T1 cells were cultured on 35 mm glass-bottom culture dishes for 24 h at 37°C. Then, 500 nM **NBS-2S-5FU** was added, meanwhile the intracellular fluorescence was tracked by a confocal laser scanning microscope (CLSM) using a 60× oil-immersion objective lens at different times (30, 60, 90, 120, and 150 min). The emission wavelength was collected from 645 to 700 nm, and the excitation wavelength was 640 nm.

2.8 *In vitro* GSH assays

3T3, 4T1, MCF7, Hela, and B16 cells were cultured on 100mm cell culture dish (Corning, #4615-ZX) at 37°C respectively and incubated until the cell confluence reached 90%. Then, collect and sonicate cells (ice bath, 200 W, sonicate for 3s, interval of 10s, repeat 30 times). Take the supernatant for GSH content testing and the GSH content was detected using Reduced Glutathione (GSH) Content Assay Kit (Solarbio, Beijing, P. R. China) according to the manufacture instruction.

2.9 Subcellular colocalization assay

4T1 and B16 cells were incubated on 35 mm glass-bottom culture dishes for 24 h at 37°C. For cellular co-localization test, **NBS-2S-5FU** (500 nM) was added to the cells and incubated for 90 min, then the cells were further stained by LysoTracker Green DND 26 (100 nM), MitoTracker Green FM (100 nM) or Hoechst 33342 (100 nM). Next, cells were visualized with laser confocal microscopy. The excitation wavelength for **NBS-2S-5FU** was 640 nm, while the excitation wavelength for LysoTracker Green DND 26 and MitoTracker Green FM were 488 nm, for Hoechst 33342 was 405 nm. The emission wavelength was collected from 645 to 700 nm for **NBS-2S-5FU**, 500 to 540 nm for Lyso Tracker Green and Mito Tracker Green, and 440 to 480 nm for Hoechst 33342.

2.10 Intracellular ROS detection

Reactive Oxygen Species Assay Kit was used according to the manufacture instruction. 4T1 and B16 cells were incubated with **NBS-2S-5FU** (0.5 μM) or NBS (0.5 μM) for 90 min followed by incubation with 10 μM DCFH-DA for another 30 min. The cells were washed with PBS and then cells treated with **NBS-2S-5FU** or NBS were irradiated with 660 nm red LED light for 5 min at a power density of 20 mW/cm^2. Then, confocal luminescence imaging was performed (excited at 488 nm, monitored at 500-540 nm).

When conducting ROS quenching experiments, 0.5 mM vitamin C (Vc) is pre-incubated for 1 h before adding drugs.

2.11 Intracellular superoxide anion radical ($O_2^{-\bullet}$) detection

4T1 and B16 cells were incubated with **NBS-2S-5FU** (0.5 μM) or NBS (0.5 μM) for 90 min followed by incubation with 10 μM DHE for another 30 min. The cells were washed with PBS and then cells treated with **NBS-2S-5FU** or NBS were irradiated with 660 nm red LED light for 5 min at a power density of 20 mW/cm^2. The fluorescence (if any) was then monitored using a confocal laser scanning microscope (CLSM) (excited at 488 nm, monitored at 570-630 nm).

When conducting $O_2^{-\bullet}$ quenching experiments, 0.5 mM vitamin C (Vc) is pre-incubated for 1 h before adding liposomes.

2.12 Cell viability assay (MTT assay)

The cell inhibiting behaviors of **NBS-2S-5FU**, 5-FU acid, and NBS for cells (3T3, 4T1, MCF-7, Hela, B16) were determined in the same procedure. Therefore, the cell viability assay was performed by 3-(4,5-dimethyl-2-thiazolyl)-2,5-diphenyl-2-H-tetrazolium bromide (MTT) method. Briefly, cells (4000-5000 cells per well) were evenly plated into 96-well plates and incubated overnight. Then cells were incubated with **NBS-2S-5FU**, 5-FU acid, and NBS. Irradiation (λ = 660 nm, power density = 20 mW/cm^2, 300 s) was imposed to light irradiation groups after 90 min incubation of **NBS-2S-5FU** or NBS. After continuing to incubate overnight, the medium in each well was replaced with fresh culture medium containing 0.75 mg/mL MTT. The plates were incubated for additional 4 h, allowing viable cells to reduce the yellow tetrazolium salt (MTT) into dark blue formazan crystals. Finally, 200 μL DMSO was added to dissolve the formazan crystals. Shaking the plate for 5 min and the absorbance at 490 nm of each well was determined by a microplate reader (SpectraMax M2e, Molecular Devices Inc. U. S. A.).

2.13 Dead/live cell co-staining

4T1 cells were cultured on 35 mm glass-bottom culture dishes for 24 h at 37°C, then exposed to different following treatments: 1) 4T1 cells untreated (Control Dark), 2) 4T1 cells irradiated with 660 nm for 5 min at a power density of 20 mW/cm^2 (Control Light), 3) 4T1 cells incubated with **NBS-2S-5FU** (0.5 μM) for 90 min at 37°C (**NBS-2S-5FU** Dark), 4) 4T1 cells incubated with **NBS-2S-5FU** (0.5 μM) for 90 min at 37°C and irradiated with 660 nm for 5 min at a power density of 20 mW/cm^2 (**NBS-2S-5FU** Light), 5) 4T1 cells co-incubated with **NBS-2S-5FU** (0.5 μM) and Vc (0.5

mM) for 90 min at 37°C (**NBS-2S-5FU**+Vc Dark), 6) 4T1 cells co-incubated with **NBS-2S-5FU** (0.5 μM) and Vc (0.5 mM) for 90 min at 37°C and irradiated with 660 nm for 5 min at a power density of 20 mW/cm^2 (**NBS-2S-5FU**+Vc Light). After different treatments, Calcein AM and Propidium Iodide co-staining were performed according to the manufacture instruction. The excitation wavelength was 488 nm, and emission wavelength was collected from 505 to 545 nm for green channel and from 600 to 700 nm for red channel.

2.14 Agarose gel electrophoresis assay

4T1 cells were seeded in 10 cm cell culture dish (Nest) and cultured with medium containing 5-FU acid (500 nM), NBS (500 nM) or **NBS-2S-5FU** (500 nM) respectively. After 90 min of incubation at 37°C in the dark, the cells in light groups were irradiated with 660 nm red LED light for 5 min at a power density of 20 mW/cm^2. After continuing to incubate for 4 h, the cell DNA was purified using Universal Genomic DNA Purification Mini Spin Kit (Beyotime) according to the manufacture instruction. The purified DNA was mixed with the DNA Loading Buffer (6X), and then subjected to agarose gel electrophoresis.

2.15 γ-H2AX immunofluorescence assay

4T1 cells were incubated on 35 mm glass-bottom culture dishes for 24 h at 37°C. Then cells were treated with following different treatments: 1) PBS, 2) NBS (500 nM), 3) **NBS-2S-5FU** (500 nM), 4) **NBS-2S-5FU** (500 nM) with 660 nm light irradiation. After cells were washed with PBS, the cells were treated according to the DNA Damage Assay Kit (Beyotime). The confocal fluorescence imaging was performed and images were collected. The excitation wavelength of DAPI and γ-H2AX were 405 nm and 488 nm, and emission signals were collected from 415 to 485 nm with blue fluorescence and from 500 to 545 nm with red fluorescence, respectively.

2.16 Annexin V-FITC/PI co-staining assay

4T1 cells were cultured on 35 mm glass-bottom culture dishes for 24 h at 37°C, then exposed to different following treatments: 1) 4T1 cells untreated (Control), 2) 4T1 cells incubated with NBS (0.5 μM) for 90 min at 37°C (NBS Dark), 3) 4T1 cells incubated with **NBS-2S-5FU** (0.5 μM) for 90 min at 37°C (**NBS-2S-5FU** Dark), 4) 4T1 cells incubated with **NBS-2S-5FU** (0.5 μM) for 90 min at 37°C and irradiated with 660 nm for 5 min at a power density of 20 mW/cm^2 (**NBS-2S-5FU** Light). After different treatments, the cell apoptosis was detected using Annexin V-FITC Apoptosis Detection Kit (Beyotime) according to the manufacture instruction. The excitation wavelength was 488 nm, and emission wavelength was collected from 505 to 545 nm for green channel and from 600 to 700 nm for red channel.

2.17 Western blot assay

4T1 cells were plated onto 10 cm cell culture dish and cultured with medium containing **NBS-2S-5FU**. The **NBS-2S-5FU**-Light group was irradiated with 660 nm LED lamp at a power density of 20 mW/cm^2 for 5 min and continued growing for 3 h. After that, cells were washed with pre-

cooled PBS three times, and lysed by RIPA lysis buffer with protease and phosphatase inhibitor on ice for 30 min. The cell debris in the resulting lysates was then removed via centrifugation at 12,000 rpm for 15 min, and the supernatants were assessed with a BCA kit to quantify proteins in them. Equal amounts of protein were added to each lane of SDS-PAGE gel for electrophoresis, and blotted onto polyvinylidene fluoride (PVDF) membranes. After blocking by protein free rapid blocking buffer at room temperature for 0.5 h, and further incubated in the presence of the primary antibody (Cleaved Caspase-3, Bcl-2, BAX, β-actin) at 1:1,000 dilution overnight at 4˚C. The membranes were then washed with TBST and incubated with secondary antibodies at 1:1,000 dilution next day. The protein expression levels were detected.

2.18 Preparation of L@NBS-2S-5FU

L@NBS-2S-5FU was prepared per the method in our previous study.[4] Briefly, DPPC, cholesterol, DSPE-mPEG2k, DSPE-PEG-FA (MW:2000) and **NBS-2S-5FU** (80:10:10:1:1) (w/w) were dissolved in 20 mL chloroform ($CHCl_3$), and then the solvent was removed by reduced pressure distillation. Later, the dried mixture was placed into a vacuum-drying oven, desiccated overnight at 35 °C, and was re-distributed in 20 mL PBS (pH 7.4). After multigelation five times in the -80 °C refrigerator, the obtained solution was then circulated under a high-pressure homogenizer 30 times and quickly percolated with 400 and 220 nm polycarbonate filter using an extruder a in water bath at 60 °C. Finally, the final product was stored at 4 °C.

2.19 *In vivo* antitumor evaluation

Female Balb/c mice (about 6 weeks aged) were purchased from Liaoning Changsheng biotechnoiogy Co. Experiments on mice were performed in accordance with the Guide for the Care and Use of Laboratory Animals published by the National Institutes of Health, all manipulations were followed by USA National Research Council regulation. This animal protocol was approved by the local scientific research ethics review committee of the Animal Ethics Committee of Dalian University of Technology (certificate number: Ethics approval number is 2018-043).

For tumor model establishment, 4T1 cells (1×10^6) were injected subcutaneously (subQ) into the right armpit to establish the 4T1 tumor model of Balb/c mice. About 7 days after inoculation, the tumor volume of about 90-100 mm^3 could be used for experiments.

For *in vivo* tumor accumulation, **NBS-2S-5FU** (100 μL, 0.2 mg kg^{-1}) and **L@NBS-2S-5FU** (100 μL, 0.2 mg kg^{-1}) was injected into tumor bearing mice via the tail veins, and fluorescence imaging was observed at different post-injection time in a I*n Vivo* Imaging System (IVIS). The excitation wavelength was 630 nm, and the collected emission wavelength was 680-720 nm. After injected for 4 hours, the mice were sacrificed and dissected to obtain the tumor tissue and major organs such as heart, liver, spleen, lung and kidneys. All tissues and organs were imaged in the same field of view in IVIS.

For PDT evaluation *in vivo*, twenty-eight tumor-bearing mice (tumor volume > 100 mm^3) were randomly divided into seven groups and performed with the following different treatments:

1) tail vein injection of PBS (100 μL) only

2) after tail vein injection of PBS (100 μL) for 4 h, light was applied to the tumor sites (660 nm, 100 mW/cm^2, 20 min)

3) tail vein injection of 5-FU acid (100 μL, 0.2 mg kg^{-1}) only

4) tail vein injection of **NBS-2S-5FU** (100 μL, 0.2 mg kg^{-1}) only

5) after tail vein injection of **NBS-2S-5FU** (100 μL, 0.2 mg kg^{-1}) for 4 h, light was applied to the tumor sites (660 nm, 100 mW/cm^2, 20 min)

6) tail vein injection of **L@NBS-2S-5FU** (100 μL, 0.2 mg kg^{-1}) only

7) after tail vein injection of **L@NBS-2S-5FU** (100 μL, 0.2 mg kg^{-1}) for 4 h, light was applied to the tumor sites (660 nm, 100 mW/cm^2, 20 min)

The day of injection was taken as day 0 of the treatment period. The tumor volume and body weight of all mice were measured every two days in each group of mice for fourteen days during the treatment period. The greatest longitudinal diameter (length) and the greatest transverse diameter (width) were used to calculate the tumor volume.

$$\text{Tumor volume} = \text{width} \times \text{width} \times \text{length}/2.$$

Moreover, after 14 days post-treatment, the mice were euthanized, tumor tissues and main organs of mice including heart, liver, spleen, lung, kidneys were harvested for histological analysis by means of hematoxylin-eosin (H&E) staining. In order to evaluate the detailed mechanism underlying **NBS-2S-5FU** anti-tumor performance, tumors were harvested after the treatment was completed and for immunofluorescence imaging of Ki67 and TUNEL investigation.

3 HRMS, ^{1}H and ^{13}C NMR spectra

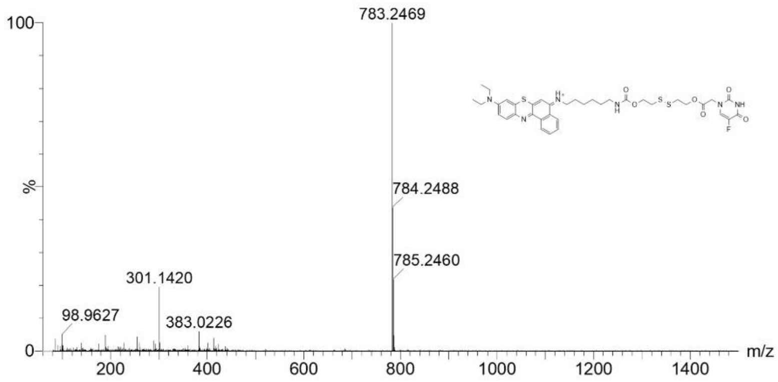

Figure S16. HRMS (ESI) of **NBS-2S-5FU**.

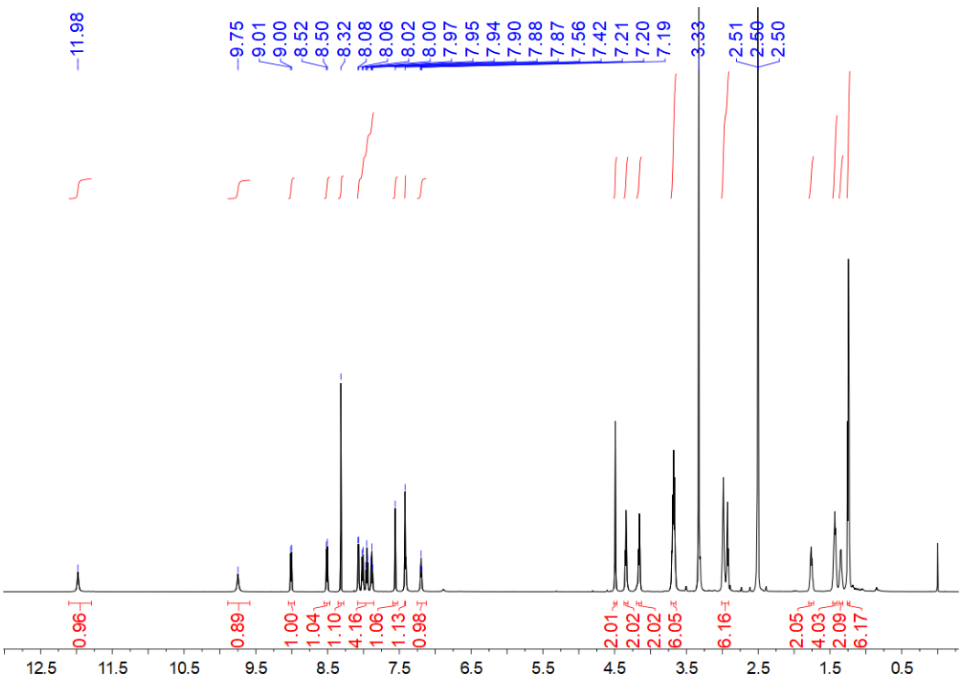

Figure S17. ^{1}H-NMR (600 MHz, d_6-DMSO) of **NBS-2S-5FU**.

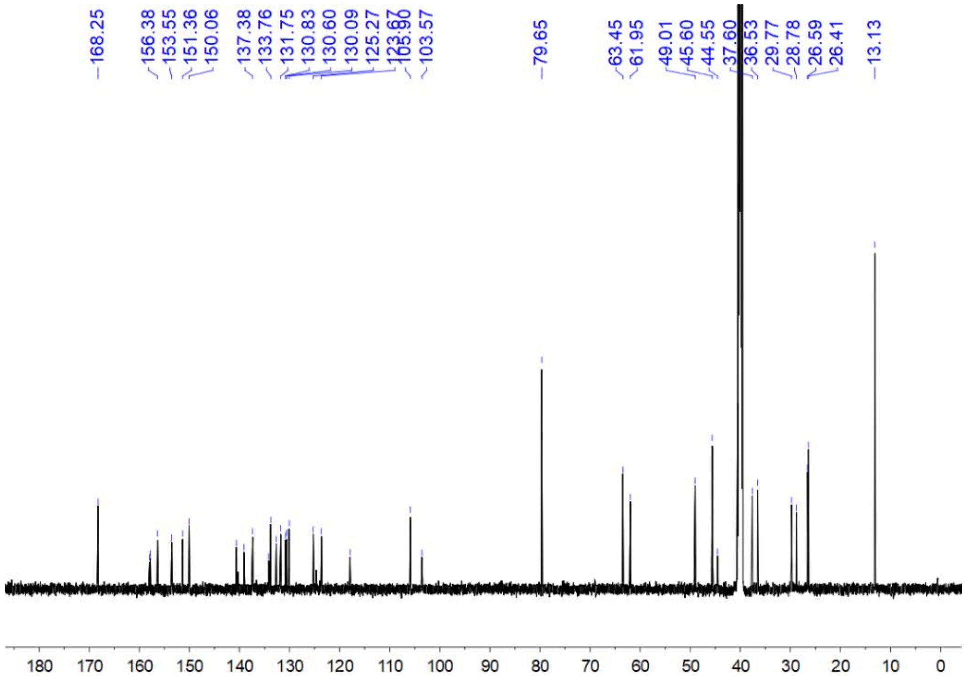

Figure S18. ^{13}C-NMR (151 MHz, d_6-DMSO) of **NBS-2S-5FU**.

4 References

[1] Huang, D., Huang, H., Li, M., Fan, J., Sun, W., Du, J., Long, S., Peng, X., *Adv. Funct. Mater.* **2022**, *32*, 2208105.

[2] Weimin, S., Gen, Z., Guifu, D., Yunxiao, Z., Jin, Z., Jingchao, T., *Bioorg. Med. Chem.* **2008**, *16*, 5665-5671.

[3] Xi, Y.-H., Yan, X., Bigdeli, F., Zhang, Q., Esrafili, L., Hanifehpour, Y., Zhang, W.-B., Hu, M.-L., Morsali, A., *Appl Organomet Chem* **2022**, *36(1)*, e6458.

[4] Shi, C., Li, M., Zhang, Z., Yao, Q., Shao, K., Xu, F., Xu, N., Li, H., Fan, J., Sun, W., Du, J., Long, S., Wang, J., Peng, X., Biomaterials **2020**, *233*, 119755.
